# Clinical significance of elevated B-type natriuretic peptide in patients with acute lung injury with or without right ventricular dilatation: an observational cohort study

**DOI:** 10.1186/2110-5820-1-18

**Published:** 2011-06-13

**Authors:** Magda Cepkova, Vineet Kapur, Xiushui Ren, Thomas Quinn, Hanjing Zhuo, Elyse Foster, Michael A Matthay, Kathleen D Liu

**Affiliations:** 1 Cardiovascular Research Institute, University of California, San Francisco, CA, 94143, USA; 2 Department of Medicine, University of California, San Francisco, CA, 94143, USA; 3 Department of Anesthesia, University of California, San Francisco, CA, 94143, USA; 4 Adult Echocardiography Laboratory, University of California, San Francisco, CA, 94143, USA

## Abstract

**Background:**

The primary objective of this study was to examine levels of B-type natriuretic peptide (BNP) in mechanically ventilated patients with acute lung injury and to test whether the level of BNP would be higher in patients with right ventricular dilatation and would predict mortality.

**Methods:**

This was a prospective, observational cohort study of 42 patients conducted in the intensive care unit of a tertiary care university hospital. BNP was measured and transthoracic echocardiography was performed within 48 hours of the onset of acute lung injury. The left ventricular systolic and diastolic function, right ventricular systolic function, and cardiac output were assessed. BNP was compared in patients with and without right ventricular dilatation, as well as in survivors versus nonsurvivors.

**Results:**

BNP was elevated in mechanically ventilated patients with acute lung injury (median 420 pg/ml; 25-75% interquartile range 156-728 pg/ml). There was no difference between patients with and without right ventricular dilatation (420 pg/ml, 119-858 pg/ml vs. 387 pg/ml, 156-725 pg/ml; *p *= 0.96). There was no difference in BNP levels between the patients who died and those who survived at 30 days (420 pg/ml, 120-728 pg/ml vs. 385 pg/ml, 159-1070 pg/ml; *p *= 0.71).

**Conclusions:**

In patients with acute lung injury the level of BNP is increased, but there is no difference in the BNP level between patients with and without right ventricular dilatation. Furthermore, BNP level is not predictive of mortality in this population.

## Introduction

B-type natriuretic peptide (BNP) has been shown to be useful for the diagnosis of congestive heart failure (CHF) in patients presenting with acute dyspnea [1]. In patients with CHF, BNP levels correlate with ventricular filling pressures and predict adverse outcome [2,3]. Similarly, BNP is elevated in patients with right ventricular (RV) dysfunction secondary to pulmonary hypertension and pulmonary embolism [4-6].

In critically ill patients with respiratory failure that requires intubation and mechanical ventilation, the diagnostic accuracy of BNP is less well established, and the role of BNP in the evaluation of increased left and right ventricular filling pressures in this setting is unclear. In patients with shock, BNP level was not shown to distinguish reliably between cardiogenic and septic etiologies or to correlate with hemodynamics but was shown to be a predictor of mortality [7].

In patients with hypoxic respiratory failure due to pulmonary edema, several recent studies have examined the utility of BNP to distinguish patients with cardiogenic pulmonary edema from patients with acute lung injury (ALI) [8-11]. These studies demonstrated that BNP levels were higher in patients with cardiogenic pulmonary edema compared with those with ALI but that the diagnostic utility of BNP was limited because of significant overlap. Furthermore, there was no correlation between BNP and filling pressures and, except in one study, BNP has not been shown to be a predictor of mortality.

Whereas in cardiogenic pulmonary edema the increase of BNP is attributed to left ventricular (LV) pressure and volume overload, the physiologic mechanisms of increased BNP levels in patients with ALI are poorly understood. By definition, patients with ALI are characterized by normal or low left-sided filling pressures [12]. However, it is well recognized that a subset of patients with ALI develops RV hypertension and RV overload [13-15]. Thus, it is conceivable that increased BNP levels in patients with ALI is due to increased RV filling pressures or that right ventricular enlargement encroaches on the left ventricle through septal shift, causing decreased LV compliance and mild increase in LV filling pressures. Therefore, we hypothesized that BNP in mechanically ventilated patients with ALI would be higher in patients with RV hypertension, dilatation, and dysfunction.

## Methods

### Study design and patient selection

This was a prospective, observational, cohort study conducted in the intensive care unit of a tertiary care university hospital. The protocol was approved by the institutional review board, and informed consent was obtained from patients or their surrogates. All patients with ALI who were admitted to the adult intensive care unit of Moffitt-Long Hospital, University of California San Francisco between December 2004 and May 2006 were eligible for the study. Inclusion criteria were age 18 years or older, positive pressure ventilation via an endotracheal tube or tracheostomy, and diagnosis of ALI. The definition of ALI was according to the American-European Consensus Conference criteria: PaO_2_/FiO_2 _ratio < 300, acute onset bilateral infiltrates on a chest radiograph, and pulmonary artery wedge pressure < 18 mmHg, or no clinical evidence of left atrial hypertension. Patients were excluded if they had the diagnosis of ALI for more than 48 hours, known severe chronic obstructive lung disease (defined as a Forced Expiratory Volume in 1 second [FEV1] < 50% predicted, history of intubation secondary to chronic obstructive pulmonary disease, receiving home oxygen therapy or chronic systemic steroids), preexisting primary or secondary pulmonary hypertension, or a history of systolic heart failure (heart failure with left ventricular ejection fraction < 40%). Patients not expected to survive more than 6 months for other reasons than ALI (terminal cancer, end-stage liver disease with Child-Pugh score more than 12, not committed to full support) also were excluded. Of 188 eligible patients, 42 patients were enrolled who had no exclusion criteria and a surrogate was available to sign informed consent.

### Clinical data collection

The primary etiology of ALI was determined based on a detailed review of clinical history. Sepsis was defined as suspected infection and presence of at least two of the systemic inflammatory response syndrome (SIRS) criteria. Pneumonia was defined as new infiltrate on chest radiograph and presence of at least two of the following three criteria: fever (temperature > 38.3°C), leukocytosis (white blood cell count > 12,000/mm^3^), or purulent secretions. As a cause of ALI, aspiration had to be witnessed or confirmed by obtaining gastric contents from the endotracheal tube. Baseline clinical characteristics and demographic data were recorded on day 1. APACHE II scores were calculated at the time of the enrollment into the study. Physiologic and hemodynamic data were recorded on day 1 and day 3 after enrollment in the study.

### Study procedures

Standard transthoracic echocardiograms were obtained using the Siemens Acuson Sequoia (Siemens Ultrasound, Mountain View, CA) or Phillips Ultrasound 5500 (Andover, MA) ultrasound systems. All echocardiograms were reviewed by an experienced cardiologist (XR) who was blinded to clinical and hemodynamic information.

RV size was evaluated according to standard echocardiography laboratory protocol based on the recommendations of the American Society of Echocardiography [7]. Semiquantitative assessment of RV size was performed based on apical four-chamber and subcostal views. RV was categorized as normal (RV size < LV size with the cardiac apex formed by the LV and an RV area ≤0.6 of LV), mildly dilated (enlarged RV size but < LV size), moderately dilated (RV size = LV size), and severely dilated (RV size > LV size). RV systolic function was categorized qualitatively as normal, mildly reduced, moderately reduced, or severely reduced.

End-diastolic and end-systolic volumes and left ventricular ejection fraction were calculated by using the two-dimensional biplane method of discs. Cardiac output (CO) was calculated by using the standard volume flow formula (the product of LV outflow (LVOT) velocity time integral, LVOT area, and heart rate).

Patterns of LV diastolic dysfunction were based on mitral inflow E/A ratios of peak velocities at early rapid filling (E) and late filling due to atrial contraction (A) and systolic or LV diastolic dominant pulmonary venous flow using VTI. Based on previously published criteria, normal LV diastolic pattern was defined as E/A ratio of 0.75 to 1.5 and systolic dominant pulmonary venous flow. Impaired relaxation pattern (mild LV diastolic dysfunction) was defined as E/A ratio < 0.75 and systolic dominant pulmonary venous flow. Pseudonormal pattern (moderate LV diastolic dysfunction) was defined as E/A ratio of 0.75 to 1.5 and LV diastolic dominant pulmonary venous flow. Restrictive pattern (advanced LV diastolic dysfunction) was defined as E/A ratio > 1.5 and LV diastolic dominant pulmonary venous flow.

RV systolic pressure was calculated by estimating the systolic pressure gradient across the tricuspid valve using the modified Bernoulli equation [16,17] and adding this value to the right atrial (RA) pressure. RA pressure was directly measured using central venous catheter at the time of the echocardiogram. In the absence of a transpulmonic gradient, PA systolic pressure was used interchangeably with RV systolic pressure [18].

Plasma for BNP measurements was collected at the time of enrollment in tubes containing potassium EDTA and was measured by clinical laboratory personnel blinded to the clinical status of the patients. The measurement was done with a validated immunoassay (Triage; Biosite, San Diego, CA).

Dead space fraction was measured using the NICO^® ^Cardiopulmonary Management System (Novametrix, Wallingford, CT). This device uses volumetric capnography [19] to calculate the partial pressure of mixed expired CO_2_, which is then used in the Enghoff modification of the Bohr equation [20].

### Statistical analysis

Data analysis was conducted using STATA 9.0 (StataCorp, College Station, TX). BNP concentrations were expressed as median and 25-75% interquartile range (IQR). To examine the relationship between the BNP levels and other variables, the BNP levels were log-transformed to achieve normality. We used Student's *t *test for the between group comparisons. The Pearson correlation was used to examine the relation between the BNP levels and other continuous variables.

## Results

### Baseline characteristics

Of the 42 patients enrolled in the study, 19 were male and the mean age was 62 ± 17 years. Demographics, etiology of ALI and comorbidities are summarized in Table 1. Baseline physiological variables are summarized in Table 2. Of note, patients were ventilated with a low tidal volume, lung protective protocol with a target plateau pressure less than 30 cmH_2_O. BNP level was elevated in mechanically ventilated patients with ALI (median 420 pg/ml; 25-75% IQR 156-728 pg/ml).

**Table 1:** Baseline demographics and clinical characteristics of the 42 patients with acute lung injury

Clinical characteristic	Value
	
Age	62 ± 17
Sex (male)	19 (45)
	
Primary etiology of ALI/ARDS	
Pneumonia	20
Sepsis	8
Aspiration	13
TRALI	1
	
Type of admission	
Medical	28 (66)
Scheduled surgical	7 (17)
Unscheduled surgical	7 (17)
	
Underlying medical illness	
Chronic liver disease	6 (14)
Glucocorticoids	2 (5)
Coronary artery disease	6 (14)
Congestive heart failure	3 (7)
Chronic renal insufficiency	2 (5)
Metastatic cancer	1 (2)
Hematologic malignancy	2 (5)
AIDS	2 (5)
Diabetes mellitus	12 (28)

AIDS = acquired immunodeficiency syndrome; TRALI = transfusion related acute lung injury

Data are means ± standard deviations or number of patients with percentages in parentheses

**Table 2:** Baseline physiological variables of the 42 patients with acute lung injury

Baseline physiological variables	Value
APACHE II	21 ± 7
SAPS II	45 ± 14
Lung injury score	2.67 ± 0.7
Oxygenation index	10.8 ± 7
PaO_2_/FiO_2_	177 ± 80
Compliance (ml/cmH_2_O)	35 ± 9
Plateau pressure (cmH_2_O)	23 ± 4
Peak inspiratory pressure (cmH_2_O)	27 ± 5
Mean airway pressure (cmH_2_O)	15 ± 4
Positive end-expiratory pressure (cmH_2_O)	9.7 ± 3.6
Tidal volume (ml)	441 ± 99
Tidal volume per kg IBW (ml/kg)	7 ± 1.3
Dead space fraction	0.56 ± 0.1

APACHE II = acute physiology and chronic health evaluation; SAPS II = simplified acute physiology score; PaO_2_/FiO_2 _= ratio of the partial pressure of arterial oxygen and the fraction of the inspired oxygen; IBW = ideal body weight

Data are means ± standard deviations

### BNP levels and right ventricular dilatation

**Figure 1:**
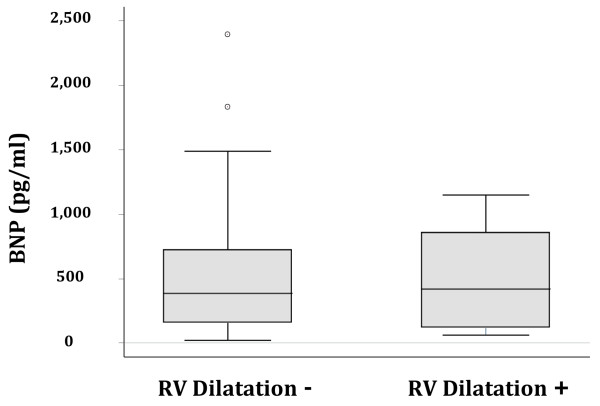
**Boxplot summary of BNP in patients with and without right ventricular (RV) dilatation**. Median levels of BNP were 387 (25-75% IQR 156-725) pg/ml in patients without RV dilation compared with 420 (25-75% IQR 119-858) pg/ml in patients with RV dilatation, which was not statistically significant (*p *= 0.96).

Right ventricular (RV) volume and systolic function was normal in 31 patients (72%), and right ventricular dilatation was present in 11 patients (26%) (Table 3). Three patients with moderate ventricular dilation also exhibited right ventricular systolic dysfunction. There was no difference in BNP between patients with and without RV dilatation (420 pg/ml vs. 387 pg/ml, *p *= 0.96; Figure 1).

**Table 3:** Hemodynamic and echocardiographic variables of the 42 patients with acute lung injury

Variable	Value
CVP (mmHg)	9.5 ± 4
SPAP (mmHg)*	42.1 ± 9.1
LVEF (%)	65 ± 7
Cardiac output (L/min)	6 ± 1.9
Cardiac index (L/min/m^2^)	3.2 ± 1
Diastolic dysfunction	
Present	18 (43)
Impaired relaxation	15 (37)
Pseudonormalization	1 (2)
Restrictive pattern	2 (4)
Not present	11 (26)
Could not be assessed	13 (31)
Tachycardia	8 (20)
Atrial fibrillation	3 (7)
Other	2 (4)
RV dilation	11 (26)
RV dysfunction	3 (7)

CVP = central venous pressure; SPAP = systolic pulmonary artery pressure; LVEF = left ventricular ejection fraction; RV = right ventricle

Data are means ± standard deviations or number of patients with percentages in parentheses

*SPAP available in 39 subjects

### BNP levels and mortality

**Figure 2:**
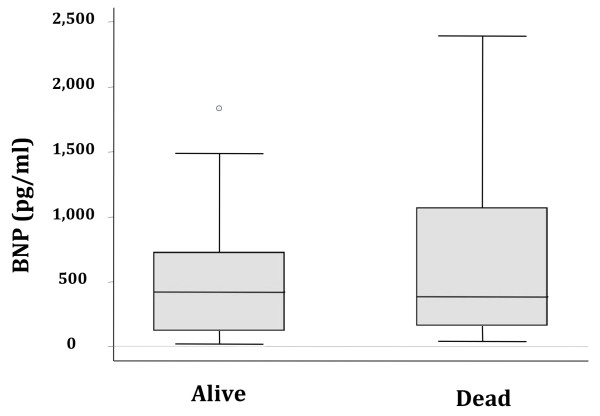
**Boxplot summary of BNP levels in survivors and nonsurvivors**. Median levels of BNP were 385 (25-75% IQR 159-1070) pg/ml in patients who survived compared with 420 (25-75% IQR 120-728) pg/ml in patients who died (*p *= 0.71).

Of the 42 patients enrolled, 15 patients died (36%) and 27 patients survived at 30 days (64%). There was no difference in BNP levels between the patients who died and those who survived (420 pg/ml vs. 385 pg/ml, *p *= 0.71; Figure 2). After stratification by renal failure (defined as a creatinine > 2 mg/dl) or shock (presence of vasopressors), BNP levels remained nondiscriminatory.

### BNP levels and relationship with other physiologic variables

There was a modest correlation between BNP levels and APACHEII (r = 0.38, *p *= 0.01) and SAPSII (r = 0.35, *p *= 0.03). There was a moderate negative correlation between heart rate and BNP levels (r = -0.35, *p *= 0.03), but there was no correlation between BNP levels and cardiac output, cardiac index, ejection fraction, systolic pulmonary artery pressure, or central venous pressure. There also was no relationship between BNP levels and net fluid balance for the previous 24 h and 8 h. Furthermore, there was no correlation with pulmonary physiologic variables, including PaO_2_/FiO_2 _ratio, oxygenation index, pulmonary compliance, and level of PEEP or lung injury score with BNP. However, there was a moderate correlation between BNP levels and pulmonary dead space fraction (r = 0.39, *p *= 0.01).

## Discussion

In this study, the levels of plasma BNP in patients with early ALI were modestly elevated and the range of distribution was wide. However, there was no difference in BNP levels in patients with or without RV dilatation or dysfunction and no relationship between BNP and mortality.

Increased levels of plasma BNP in patients with ALI/ARDS have been previously reported by other authors in several observational studies [8-11]. However, it is not clear what pathophysiological mechanisms are primarily responsible for the increased BNP levels in this patient population. Pulmonary hypertension causing right heart strain, leading to release of BNP from the right ventricular myocardium has been the most commonly implicated mechanism [21,22]. Several other mechanisms have been proposed. Hypoxia has been shown to increase cardiac gene expression of BNP [23,24] and decrease lung expression of the NPR-C clearance receptor leading to increased plasma levels of BNP in animal models [25]. Transcription of the BNP gene has been described not only in cardiac myocytes but also in the lung [26]. Thus, it has been suggested that BNP is released in lung tissue in response to pulmonary capillary leakage [27].

Pulmonary hypertension with RV dysfunction is a well-recognized complication of ALI in mechanically ventilated patients [28-30]. The incidence of cor pulmonale, historically documented to be up to 60% [14], has decreased with the introduction of low tidal volume lung-protective ventilation, but it is still reported to be approximately 25% in an article published in 2001 [31]. There is evidence from other patient populations to support the hypothesis that elevated BNP levels in patients with ALI are caused by RV strain. In patients with isolated RV dysfunction due to variety of conditions, BNP levels have been shown to be elevated. For example, patients with chronic respiratory failure who develop cor pulmonale have significantly higher BNP levels compared with patients with chronic respiratory failure without cor pulmonale or controls [32,33]. In patients with idiopathic pulmonary hypertension, BNP was elevated and was correlated with the severity of RV dysfunction and outcome [5,6]. Similar relationships have been demonstrated in patients with pulmonary embolism complicated by RV dysfunction, where BNP levels were significantly higher and predictive of mortality [4,34,35].

However, in contrast to those findings, our study showed no difference in the plasma levels of BNP in patients with or without RV dilatation. Furthermore, there was no correlation between systolic pulmonary artery pressure and BNP levels. The different findings may be explained by the timing of measurements obtained. Pulmonary hypertension with subsequent RV dilatation and dysfunction in mechanically ventilated patients with ALI is a result of a combination of factors. These include abnormalities of pulmonary blood flow due to formation of microthrombi in the pulmonary vasculature, hypoxemic vasoconstriction, and positive end-expiratory pressure. In our study, BNP levels and echocardiographic measurements were performed early in the course of the disease (as soon as possible after the diagnosis of ALI was made), thus potentially minimizing the effect of these factors on BNP levels, pulmonary artery pressures, and RV geometry and function. However, although the systolic pulmonary artery pressures were significantly elevated and BNP levels were markedly elevated, there was no relationship between these two variables. Additionally, BNP did not correlate with RV dilatation. Thus, our study suggests that BNP elevation in the early stages of ALI may not be caused by RV strain alone.

BNP has been established to be a predictor of mortality in a variety of chronic and acute conditions, including congestive heart failure, coronary artery disease, acute coronary syndromes [36,37], and acute pulmonary embolism [4]. In critically ill patients, the prognostic value of elevated BNP is less clear. In several studies, BNP has been predictive of outcome in patients with cardiogenic and septic shock [7,38,39]. However, in a mixed population of patients who present with severe sepsis and septic shock, the results are inconsistent; some studies have shown BNP to be predictive of mortality [40], others have not [41]. Similarly, in patients presenting with hypoxic respiratory failure due to CHF or ALI, the studies have shown conflicting results. Jefic et al. [9] showed no relationship of BNP with mortality in 41 critically ill patients with respiratory failure (909 ± 264 in survivors vs. 841 ± 171 in nonsurvivors). Rana et al. [42] in a study of 204 patients who presented with pulmonary edema found that BNP levels did not differ between survivors and nonsurvivors (median 528 vs. 774, *p *= 0.24; O. Gajic, personal communication). Our data are consistent with those findings. In contrast, in a study by Karmpaliotis et al. [10], BNP showed a strong graded relationship with mortality risk in 79 subjects admitted to the ICU with hypoxic respiratory failure. In the subgroup of patients with ALI (n = 51), this relationship did not reach statistical significance but the trend was present (*p *= 0.07). We are unable to fully explain the discrepancies between these studies, but these may be partially attributed to different patient populations, study designs, and statistical analyses. We found interesting that Karmpaliotis et al. elected to analyze the mortality data using tertiles of BNP; however, using this method to analyze our data did not change our results. Also, in their study, 52% of the patients with ALI were in shock, and BNP has been shown to predict mortality in patients with shock. Because the authors did not stratify for the presence of shock, it is possible that shock could have accounted for the significant relationship with mortality.

RV dysfunction has been associated with an increased risk of death in patients with ALI [43-46]. In our study, we did not find a relationship between RV dilatation as a measure of RV dysfunction and mortality. However, compared with other studies that have shown this association, our study was modest in size. Furthermore, in addition to receiving lung protective ventilation, our patients also received relatively "RV protective" ventilation, as has been suggested by Bouferrache and Vieillard-Baron [46]. Specifically, our patients received protocolized low tidal volume ventilation with a target plateau pressure of 30 cmH_2_O or less and relatively low PEEP (following the protocol described in [47]) and had minimal hypercapnia (only one subject had a paCO_2 _> 50 mmHg). Thus, perhaps the impact of ALI on RV dysfunction and associated mortality was reduced by our overall ventilatory approach, despite the fact that the ventilatory approach was not specifically modified after the detection of RV dysfunction by echocardiography in a protocolized fashion in this study.

The strength of our study includes its prospective design, rigorous collection of clinical and hemodynamic variables, and blinded interpretation of echocardiograms. However, some limitations should be mentioned. First, because our study was single-center and prospective, the sample size was modest and may limit our conclusions. Second, we used only a single measurement of BNP. Both echocardiography and BNP levels were obtained as soon as feasible after the diagnosis of ALI and every effort was made to coordinate these measurements. Because BNP has half-life of approximately 20 minutes and is known to fluctuate with changes in loading conditions, serial measurements of BNP may have been more useful. However, previous studies have shown that daily BNP levels in ICU patients do not change significantly [11,41].

In summary, in patients with acute lung injury the plasma levels of BNP are increased, yet the reasons for this increase remain unclear. In this study, BNP levels were elevated regardless of right ventricular dilatation or dysfunction and an elevated BNP level was not predictive of mortality in this population of patients with ALI.

## Conclusions

The diagnostic utility of BNP is not well established in critically ill patients with hypoxemic respiratory failure attributed to ALI. We examined the association of BNP levels with RV dilatation and with patient outcomes (mortality) in patients with ALI. Although BNP levels were elevated in patients with ALI, there was no association with RV dilatation or mortality in our prospective cohort study. Therefore, BNP seems to have limited diagnostic utility in this context.

## Competing interests

The authors declare that they have no competing interests.

## Authors' contributions

MC was responsible for the design and execution of the study, including screening and consenting eligible study subjects, data collection (including echocardiography measurements), data analysis, and manuscript preparation. VK and TQ were involved in screening and consenting eligible study subjects and data collection. XR and EF were responsible for interpretation of the echocardiography results. HZ was responsible for database management and data analysis. MAM was responsible for study design, data analysis, and manuscript preparation. KDL contributed to data analysis and manuscript preparation and revision.
